# Measuring diagnostic performance of COVID-19 tests: lessons for the next pandemic

**DOI:** 10.2807/1560-7917.ES.2021.26.45.2101050

**Published:** 2021-11-11

**Authors:** Jacob Moran-Gilad

**Affiliations:** School of Public Health, Faculty of Health Sciences, Ben Gurion University of the Negev, Beer Sheva, Israel

The coronavirus disease (COVID-19) pandemic triggered an unprecedented global response to meet the demands for laboratory testing to diagnose infections with severe acute respiratory syndrome coronavirus 2 (SARS-CoV-2) . The majority of developed assays remain commercial and build upon well-established laboratory methods, such as reverse-transcriptase quantitative PCR (RT-qPCR) for detection of virus-specific nucleic acid sequences, assays for antigen detection and assays for detection of various anti-SARS-CoV-2 antibodies. Many of the tests are defined as rapid diagnostic tests (RDT), designed for point-of-care testing [1].

To fill the diagnostic gaps in the wake of the pandemic, many diagnostic tests were developed, validated and approved for use in an expedited manner. As the pandemic progresses, there is an increasing interest in the quality control and measurement of performance of COVID-19 diagnostics. Therefore, it is not surprising that informative papers that report on the evaluation of the performance of diagnostics are increasingly emerging.

Three notable papers published over the last 2 weeks in *Eurosurveillance* address the performance of COVID-19 diagnostics. Puysknes et al. report the setup of a decentralised evaluation approach and on the technical evaluation of 31 RDT for SARS-CoV-2 antigen detection based on lateral flow [2]. Using a panel of 50 pooled clinical specimens, representing a wide range of virus concentrations, the 31 evaluated assays demonstrated considerable variation in analytical and clinical sensitivity. Most assays demonstrated acceptable sensitivity (> 80%) when applied on samples associated with more than 10^6^ genome copies and lower quantification cycle (Cq) values (≤ 25), presumed to represent an infective stage. Still, only a few performed satisfactorily at high Cq values. Using the same sample panel, Scheiblauer et al. evaluated the performance of 122 antigen RDT, of which only 79% passed the criterion of  > 75% sensitivity for panel samples with Cq ≤ 25 [3]. Several assays appeared to have failed the evaluation entirely, whereas others exhibited a very high sensitivity. Both studies not only provide valuable information with respect to the actual performance of a large number of widely distributed commercial RDT kits, but also report a feasible evaluation framework that could be widely adopted.

The paper by Van Walle et al. reports a meta-analysis of the clinical performance of commercial assays for molecular detection of SARS-CoV-2 and antibody tests [4]. The analysis was based on more than 110,000 test results reported in 151 publications or collated from 12 countries over the first 5 months of the pandemic. Varying levels of performance in terms of sensitivity and specificity were evident. Interestingly, the authors found that reports of performance by manufacturers tended to significantly overestimate actual performance, testifying for the importance of independent assessment of commercial kits.

The striking variability in the performance of different commercial assays highlights the importance of quality control and quality assurance in a pandemic situation. The rapid deployment and adoption of laboratory tests resulted in notable challenges, such as the lack of certified reference materials, measurement uncertainties, lack of epidemiological or clinical correlates, and lack of standardisation and harmonisation, to name a few. For example, a recently published study demonstrated an inter-laboratory variation of > 1,000 in virus copies/mL when comparing Cq values, suggesting that widely used units such as the Cq (also known as C_t_) that are considered indicative of infectiousness are not standardised and could prove misleading [5]. Moreover, when recommended cut-offs (e.g. Cq ≤ 25) were applied, the sensitivity of molecular diagnostics dropped considerably. In addition, the distribution of viral loads across patient populations (reflecting the epidemiological situation) appears to have a substantial effect on the accuracy of measures such as the Cq values. While the analytical performance of RT-qPCR could be controlled using proper methodologies, other components of molecular testing are much more challenging to evaluate and standardise, such as the adequacy and reproducibility of sample collection (sample source and collection technique), the impact of sample storage, transport and pre-treatment, and variations on the molecular detection process such as sample pooling. With RDT, even greater challenges exist because the tests are performed in an uncontrolled environment and commonly by non-professional operators.

While setting performance standards is paramount for ensuring standardisation and harmonisation, we must consider, based on experience accumulating during the COVID-19 pandemic response, that testing requirements could differ between settings and applications. For example, a test used for triaging patients with suspected COVID-19 in the hospital may necessitate a different performance standard as compared with a 'gating' test used for ruling out SARS-CoV-2 infection in asymptomatic individuals attending public events. Similarly, serological tests used at the point of care for surveillance purposes may require different performance standards as compared to formal serology performed during a clinical work-up. 

Looking forward, the scientific and public health communities should further develop solutions in order to close the gaps identified during the global laboratory response to the COVID-19 pandemic. This requires a holistic approach that brings together a wide range of stakeholders from the medical, laboratory, public health, industry and regulatory fields. There is an urgent need for a globally accepted framework that will inform the development, validation and implementation of new assays in the face of emerging public health threats. Having fit-for-purpose solutions for ensuring test performance should be an integral part of national and global emergency preparedness to ensure a rapid and robust laboratory response to future pandemics.
